# Advances in Nanoengineered Terahertz Technology: Generation, Modulation, and Bio-Applications

**DOI:** 10.34133/research.0562

**Published:** 2025-01-13

**Authors:** Zhongwei Jin, Jing Lou, Fangzhou Shu, Zhi Hong, Cheng-Wei Qiu

**Affiliations:** ^1^ College of Optical and Electronic Technology, China Jiliang University, Hangzhou 310018, China.; ^2^ Centre for Terahertz Research, China Jiliang University, Hangzhou 310018, China.; ^3^ Innovation Laboratory of Terahertz Biophysics, National Innovation Institute of Defense Technology, 100071 Beijing, China.; ^4^ Department of Electrical and Computer Engineering, National University of Singapore, Singapore 117583, Singapore.

## Abstract

Recent advancements in nanotechnology have revolutionized terahertz (THz) technology. By enabling the creation of compact, efficient devices through nanoscale structures, such as nano-thick heterostructures, metasurfaces, and hybrid systems, these innovations offer unprecedented control over THz wave generation and modulation. This has led to substantial enhancements in THz spectroscopy, imaging, and especially bio-applications, providing higher resolution and sensitivity. This review comprehensively examines the latest advancements in nanoengineered THz technology, beginning with state-of-the-art THz generation methods based on heterostructures, metasurfaces, and hybrid systems, followed by THz modulation techniques, including both homogeneous and individual modulation. Subsequently, it explores bio-applications such as novel biosensing and biofunction techniques. Finally, it summarizes findings and reflects on future trends and challenges in the field. Each section focuses on the physical mechanisms, structural designs, and performances, aiming to provide a thorough understanding of the advancements and potential of this rapidly evolving technology domain. This review aims to provide insights into the creation of next-generation nanoscale THz devices and applications while establishing a comprehensive foundation for addressing key issues that limit the full implementation of these promising technologies in real-world scenarios.

## Introduction

The terahertz (THz) frequency range, spanning from 0.1 to 10 THz, sitting between the microwave and infrared (IR) regimes, holds immense potential across a wide range of applications [1–3]. This previously overlooked region of the electromagnetic spectrum has already found its significant use in spectroscopy [3,4], imaging [5,6], high-speed communication [7,8], biomedical diagnostics [9,10], and more. The unique ability of THz waves to penetrate optically opaque materials, interact with vibrational and rotational modes of molecules, and provide label-free sensing of biological interactions makes this technology a pioneer in fields ranging from material science to bioscience.

Recent advances in nanotechnology have deeply influenced the evolution of THz science and technology [10–13]. The capability of fabricating structures at the nanoscale has led to the creation of compact, table-top highly efficient THz devices with superior performance characteristics. Nanoscale engineering provides precise control over material properties and device architectures, enabling the generation and modulation of THz waves with unprecedented efficacy. These advancements have resulted in significant enhancements in THz imaging [5,6,14,15], spectroscopy [3,4,16–18], and especially bio-applications, such as biosensing [19–22] and biofunction [23–28] techniques, offering higher resolution, increased sensitivity, and broader bandwidths.

The generation of THz radiation has garnered significant interest due to its broad applications in imaging, communication, and spectroscopy. Various techniques have emerged for the efficient production of THz radiation, harnessing advances in nanoengineering. Photonic approaches, such as photo-induced THz emission using femtosecond lasers, exploit the ultrafast dynamics of charge carriers to create THz pulses [29–32]. Plasmonic devices, leveraging surface plasmon resonances, enhance THz field generation through localized electromagnetic fields that facilitate nonlinear processes [4,33–35]. Quantum cascade lasers (QCLs) offer compact, semiconductor-based THz sources operating via intersubband electronic transitions in quantum wells [36–39]. These methods highlight the synergy between nanotechnology and THz generation, paving the way for more efficient and versatile THz sources essential for technological advancement. Through leveraging nanostructured heterostructures [21,29,30,40,41], metasurfaces [11,42–52], and hybrid material systems [44,53,54], researchers can achieve unprecedented control over the amplitude, phase, and polarization at the point of generation, with an ultrabroadband bandwidth that spans almost the entire THz regime and even extends to the IR. These innovations are crucial for applications such as high-resolution imaging and spectroscopy, and wireless communications, yet integrating these advancements into functional devices poses new questions regarding scalability, efficiency, and reliability. Similarly, breakthroughs in THz modulation techniques, ranging from homogeneous modulation including amplitude [55,56], phase [57,58], and polarization [53,59] modulators to spatial light modulators [6,14], now enable precise control over THz properties, but the intricate interplay between metasurfaces and active materials offers new challenges and opportunities for optimization.

Moreover, the intersection of THz technology with biological systems represents one of the most exciting frontiers in this field. Since the absorption peaks of pivotal substances in biological activities, such as molecular vibrations and rotational modes, consistently fall within the THz regime [19,60,61] and extend into the IR [16,18,24], THz and IR waves have garnered significant interest for applications in biosensing [62–66], biofunction modulation [23,25,27], and noninvasive bio-imaging [67]. For example, it is reported that target biomaterials like virus and cells can be quantitatively categorized with respect to their subtypes in the THz spectrum based on refractive index and absorption coefficient [68]. Compared with traditional magnetic resonance imaging and x-ray imaging, THz radiation accompanying with nondestructive properties has been demonstrated to diagnose tumor tissues, skin cancers, and brain glioma [69–71].

Previous reviews have reported on the developments in THz wave generation [3,72–74], THz wave modulation [13,75,76], and THz biological applications [10,12,60,67] in a separate manner. Additionally, many reviews have concentrated on the design of micro- and nanostructured materials and metasurfaces [12,42,51,77]. With the rapid progress of THz nanoengineered technology, nano- and micro-structured THz emitters are now capable of modulating the properties of THz waves at the generation stage. The advancements in nano- and micro-sized THz bio-devices have demonstrated significant improvements in their performance. By reviewing and synthesizing these cutting-edge developments, this review aims to reveal both the opportunities and challenges that lie ahead for the further advancement of nanoengineered THz technologies. Our goal is to provide insights for researchers and engineers engaged in next-generation THz devices and applications while establishing a comprehensive foundation for addressing the critical issues that restrict the full implementation of these promising technologies in practical scenarios.

This review is structured into several key sections. Following this introduction, the generation section discusses state-of-the-art advancements in nanoengineered THz emitters based on heterostructures, metasurfaces, and their hybrid systems, with an emphasis on their physical mechanisms, structural designs, and functionalities. The modulation section then delves into various techniques for THz wave modulation, encompassing both homogeneous and individual modulation, while highlighting recent innovations and their implications. The subsequent section on bio-applications examines the intersection of THz technology with biological systems, detailing novel biosensing and biofunctional techniques. Finally, the conclusion and perspectives section summarizes the findings and reflects on future trends and challenges within the field of nanoengineered THz technology. Each section is designed to provide a comprehensive understanding of the advancements and potential of this rapidly evolving domain.

## Generation

Nanoengineered THz emitters are transforming the landscape of THz technology, offering unprecedented advantages over traditional bulky THz sources [3,73,78]. These table-top emitters, particularly those based on heterostructures, metasurfaces, and their hybrid systems, harness the unique properties of artificially designed nanoscale materials to achieve precise control over THz wave generation and manipulation simultaneously. Heterostructures, composed of layered materials with distinct electronic properties, enable efficient THz emission through advanced spintronic mechanisms in both low-energy and high-energy systems. Meanwhile, metasurfaces provide exceptional flexibility in shaping THz wavefront by exploiting resonant interactions at subwavelength scales. Recent developments have focused on optimizing material composition and structural design to enhance emission efficiency, spectral coverage, and wavefront control. This section explores these advancements, highlighting the potential of nanoengineered THz emitters to drive innovation in various technological domains.

### Heterostructures based on spintronics

THz emission from ferromagnetic (FM)/nonmagnetic metal (NM) bilayer and multilayer heterostructures has been a prominent investigation area in recent years [74]. By precisely controlling the composition, thickness, and sequence of these layers at the nanoscale, heterostructures can manipulate charge carriers and energy band structures beyond traditional bulk materials. The working mechanisms of femtosecond laser-driven THz transients are rooted in ultrafast charge and spin dynamics [79,80], including phenomena such as the inverse spin Hall effect (ISHE) [81], inverse Rashba–Edelstein effect (IREE) [82], inverse spin torque effect (ISOTE) [83], spin-galvanic effect (SGE) [84], and ultrafast spin Seebeck effect (USSE) [85]. This section highlights recent advancements on THz spintronic emitters (STEs) dominated by ISHE, which offer higher THz emission intensity (often several orders of magnitude greater), broader spectral coverage, more flexibility and scalability, and enhanced robustness.

**Fig. 1.**
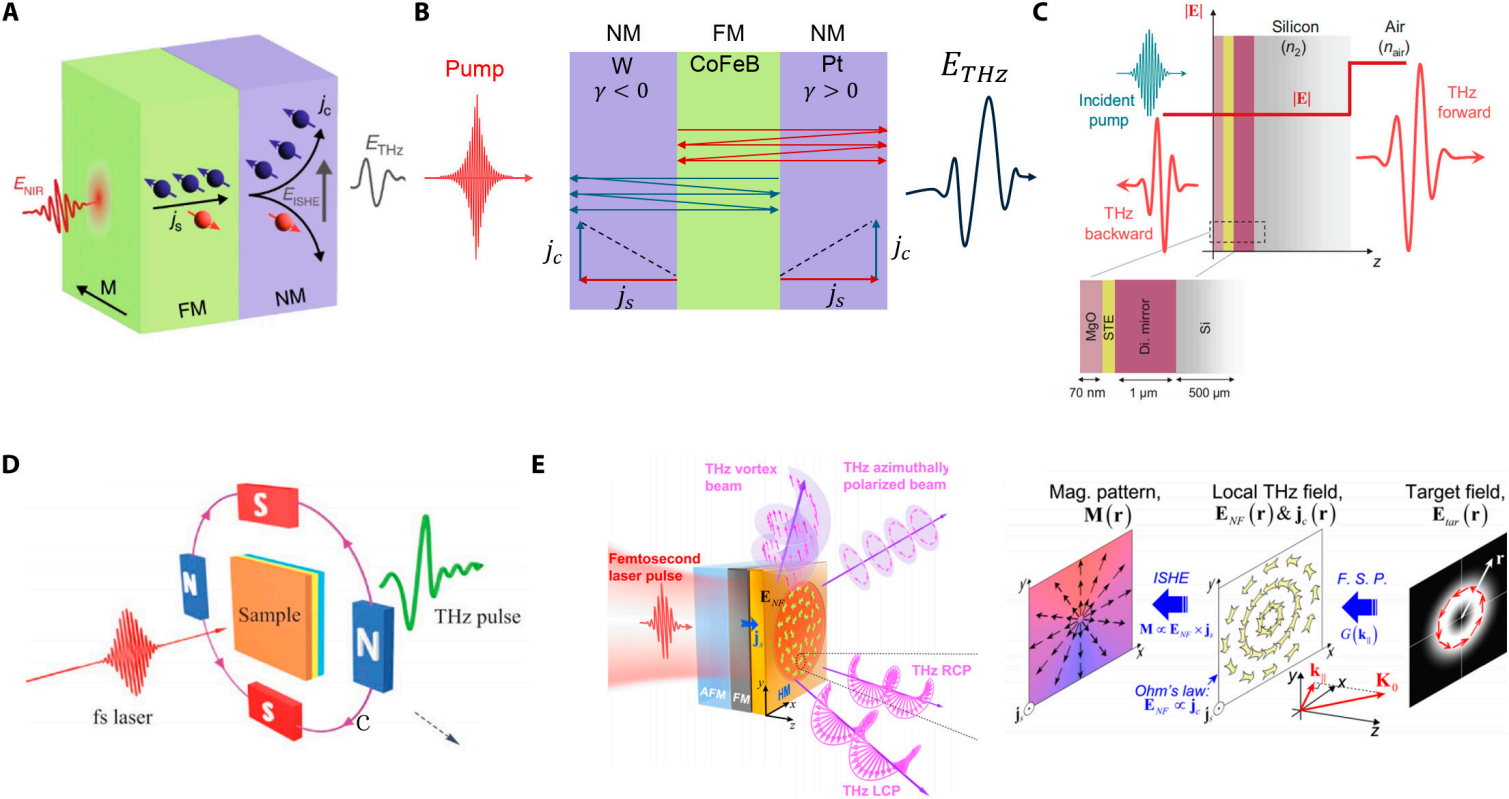
THz generation and manipulation from heterostructures. (A) THz generation from an FM(Fe)/HM(Au or Ru) bilayer heterostructure. (B) THz generation from an HM(W)/FM(CoFeB)/HM(Pt) trilayer heterostructure. (C) THz generation from a Si-based heterostructure developed from the W/CoFeB/Pt heterostructure. (D) Polarization manipulation of a typical STE (W/CoFeB/Pt) via external magnetic field distribution. (E) Structured THz generation and manipulation from an AFM(IrMn_3_)/FM(Fe_21_Ni_79_)/NM(Pt) trilayer heterostructure. (A) Reprinted with permission from [73]. Copyright 2023 Springer Nature. (C) Reprinted with permission from [87]. Copyright 2023 American Physical Society. (D) Reprinted and adapted with permission from [72,88]. Copyright 2022 Springer Nature, Copyright 2019 Wiley-VCH. (E) Reprinted with permission from [89]. Copyright 2024 Springer Nature.

For ISHE-dominated STEs, heavy metals (HMs) are preferred for the NM layer due to their strong spin–orbit coupling, which significantly promotes the efficiency of the ISHE [86]. In 2013, Kampfrath et al. [29] laid the groundwork of ultrafast broadband THz pulses generation via spintronic effects in FM/NM heterostructures (Fig. 1A). Femtosecond pulses induce ultrafast spin currents from the FM layer into the NM layer, which converted into THz radiation through ISHE:

$$E_{THz}\propto \gamma \cdot j_{s}\times M/|M|$$ (1)

where γ is the spin Hall angle, $j_{s}$ is the spin-polarized current, M is magnetization, and the charge current is $j_{c}=\gamma \cdot j_{s}\times M/\left|M\right|$. The emitted THz wave is linearly polarized, with intensity scaling linearly with pump fluence as shown in Fig. 1A. Equation 1 indicates that the amplitude, phase, and polarization of the emitted THz field can be tuned via the material’s spin Hall angle and external magnetization. The polarization maintains perpendicular to the FM layer's magnetization and independent of pump polarization. In this work, the proposed FM (Fe)/NM (Au) sample produces a field amplitude 2 orders of magnitude smaller than that of a 300-mm-thick ZnTe crystal, covering a frequency range from 0.3 to 20 THz, which spans nearly the entire THz regime and extends into the IR. Enhancing conversion efficiency involves optimizing material selection and heterostructure geometry. In 2016, Seifert et al. [30] demonstrated ultrabroadband THz radiation spanning 0.1 to 30 THz using a trilayer heterostructure (5.8-nm-thick W/Co_40_Fe_40_B_20_/Pt) as illustrated in Fig. 1B. Constructive superposition of charge currents from NM layers with opposite spin Hall angles in Pt and W contributes to enhanced THz emission. Film thickness optimization showed that the thin metal film acts as a Fabry–Pérot cavity, amplifying both pump and THz waves until reflection losses exceed bulk attenuation. Due to the attractive features of bilayer and trilayer STEs, many studies have focused on these approaches to enhance THz emission performance and explore the underlying physics.

The impact of pump light frequency on trilayer STE systems has been investigated. The near-IR (NIR) pump sources are generally employed to excite STEs due to their mature femtosecond laser technology and excellent THz detection system compatibility. Herapath et al. [90] found that THz emission efficiency from a W/CoFeB/Pt trilayer in the NIR range (900 to 1,500 nm) remained largely independent of pump wavelength. However, later studies with a Ta/NiFe/Pt heterostructure showed blue light (400 nm) producing THz transients with amplitudes nearly 3 times greater than NIR (800 nm) excitation, suggesting that higher photon energy enhances spin polarization transport [91]. Differences in wavelength ranges and materials account for these findings, indicating additional physical insights into NM/FM/NM STEs.

Efforts to refine trilayer heterostructures to further enhance THz emission include inducing surface plasmon resonance by adding gold nanorods to a W/CoFeB/Pt STE [92], increasing THz emission by 140%. Antenna-coupled STEs reported a maximum 2.42-fold increase in peak-to-peak THz signals [93]. Through inclusion of a dielectric mirror (TiO_2_ and SiO_2_ overlayers), which forms a cavity with the substrate, the THz emission amplitude can be enhanced by up to a factor of 2 covering 0 to 2 THz [90]. An upscaling scheme using a cascaded Pt/CoFe/Ta STE on a flexible polyethylene terephthalate (PET) substrate [94], which maintains phase alignment between the pump and THz emission, achieves 1.55 times the THz amplitude of a single STE/PET.

Lately, a Si-based STE, optimized for optical and thermal environments for THz generation, was proposed [87]. The scheme of the Si-based STE is illustrated in Fig. 1C. The typical Pt/CoFeB/W trilayer is embedded between a dielectric mirror ([SiO2(165 nm)|TiO2(94 nm)]5) and an impedance-matching MgO (70 nm) layer. Si is chosen as substrate for preventing excess energy accumulation. The joint effects of the design generates THz pulses with peak electric fields above 1.5 MV/cm and fluence above 1 mJ/cm^2^ for 800-nm, 5-mJ pump pulses over a frequency window of 0.1 to 11 THz, which is rivaling that of a state-of-the-art LiNbO_3_ source.

So far, heterostructure-based STEs have demonstrated their potential in linear and nonlinear THz spectroscopy. For example, transmission spectra of a 7.5-μm-thick polytetrafluoroethylene (PTFE; Teflon) sample were demonstrated using the W/CoFeB/Pt sample as shown in Fig. 1A. Linear optical response of a riboflavin sample was reported using the cascaded STE/PET assembling with a dual-torus toroidal metasurface [94]. The result aligns well with that of commercial THz time-domain spectroscopy (THz-TDS) (EKSPLA) with photoconductive switch. Also, the high field and strong fluence from the Si-based STE is sufficient to drive the nonlinear process, opening a new avenue to nonlinear THz spectroscopy [17,87,95].

While the polarization of THz pulses from heterostructure-based STEs can be tuned via external magnetization, it normally restricts to linear polarization [29,30]. Circularly polarized THz waves are crucial for studying chiral molecules and materials [96], emerging quantum materials [97], as well as advanced imaging [72] and sensing technologies [5]. Conventional methods for converting linearly polarized THz waves to circularly polarized ones often suffer from narrow bandwidth and low efficiency. A more optimal approach would be generating fully controllable polarization THz waves directly from the source.

Kong et al. [88] revisited Eq. 1 and demonstrated tunable elliptical THz emission from a standard trilayer STE by tailoring the magnetic field distribution (Fig. 1D). By applying a nonuniform, twisted magnetic field, 2 orthogonal electric field components with controllable phase differences are generated perpendicular to the local magnetic field direction in the radiated far field. The helicity of the elliptical emission is tuned by reversing the magnetic field distribution, while the azimuthal angle is controlled by rotating the magnets and heterostructure sample. Ellipticity is adjusted by varying the emitting areas to modulate the amplitude. However, precise control of the magnetic field distribution remains challenging, hindering its application in many scenarios. A cascaded scheme consisting of 2 typical W/CoFeB/Pt STEs realized the circular polarized THz emission [98]. By setting the external magnetic field direction, optimal pump fluence, and proper distance between the 1st and 2nd STE, radiation from the 2 STEs achieves nearly equal amplitude, perpendicular polarization, and a 90-degree phase shift, forming perfect circular polarized THz light.

In a latest work, Wang et al. [89] proposed an antiferromagnetism (AFM) (IrMn_3_)/FM(Fe_21_Ni_79_)/NM(Pt) heterostructure, which facilitates the structural generation of THz wave (Fig. 1E). Changing the typical NM layer into an AFM layer induces unidirectional magnetic anisotropy in the FM layer via exchange coupling with the adjacent AFM layer. This way, magnetization programming is achieved via laser-induced local heating and field cooling, which re-aligns the AFM-FM exchange coupling. By selectively altering the local magnetization pattern on the sample surface, a wide range of structured THz waves can be generated. This method allows for precise control over emitted THz waves in a broadband frequency range.

### Metasurfaces

Research on generating THz pulses using nanostructures can be traced back to over a decade ago. Polyushkin et al. [99] demonstrated THz pulse generation from silver nanoparticle arrays driven by ponderomotive acceleration of photoelectrons. Later, Keren-Zur et al. [33] reported efficient THz emission from a single 40-nm-thick layer of gold split-ring resonators (SRRs) via engineered nonlinear metasurfaces.Unlike heterostructures, where THz emission performance is primarily governed by material and geometry, metasurfaces exploit resonant phenomena to enhance local electromagnetic fields, significantly boosting the nonlinear conversion processes necessary for THz emission. Combining their unparallel control of light’s amplitude, phase, and polarization, metasurfaces offer exceptional versatility in simultaneously generating and manipulating THz radiation. The concurrent control offers advantages over traditional THz generation methods, circumventing issues such as material dispersion and phase matching. This approach opens new possibilities for THz spectroscopy [4] and imaging [43], eliminating the need for additional bulky THz optics and enabling compact, high-efficiency THz devices for advanced applications.

#### THz generation and manipulation from plasmonic metasurfaces

SRRs have been extensively employed for THz generation and manipulation due to their versatile geometric design [4,33,35,104]. They support both magnetic and electric dipole resonances, which are crucial for efficient interaction with THz waves. The intentional symmetry breaking in SRRs enhances second-order nonlinear processes such as optical rectification (OR), driving efficient THz generation. Additionally, their geometric flexibility allows for precise control of phase, amplitude, and polarization of the emitted THz waves, facilitating advanced wavefront manipulation directly at the generation stage.

Tal et al. [4] presented a plasmonic metasurface THz emitter (MTE) composed of 40-nm-thick gold SRRs optimized for localized surface plasmon resonances (LSPRs) at NIR wavelengths. THz emission stems from the strong field localization at the SRR surfaces, boosting nonlinear optical processes such as OR. Instead of using expensive amplified laser systems demonstrated in previous works, the proposed MTE can be excited through a nanojoule femtosecond pulses from a compact, low-power laser oscillator. The THz conversion efficiency is comparable to that of a 0.1-mm-thick ZnTe crystal. Spectroscopic analysis of α-lactose monohydrate powder based on the proposed system revealed a clear absorption line at 0.53 THz, implying the MTE’s ability for low-cost, compact THz spectroscopy.

Simultaneous phase and amplitude control of emitted THz pulses at the generation stage was later demonstrated through a nonlinear metasurface Fresnel zone plate (FZP) [104]. The metasurface FZP is composed of zones with inverted oriented SRRs designed to support LSPR at pump wavelength, thereby enhancing nonlinear interaction. As a result, broadband THz radiation and selectively focusing of different THz frequencies along the optical axis can be conveniently achieved. Notably, the focused radiation delivers few-cycle pulses with varying carrier frequencies due to the direct space to time mapping between the metasurface FZP and the generated pulse. This new finding holds potential for time-of-flight noninvasive imaging applications.

**Fig. 2.**
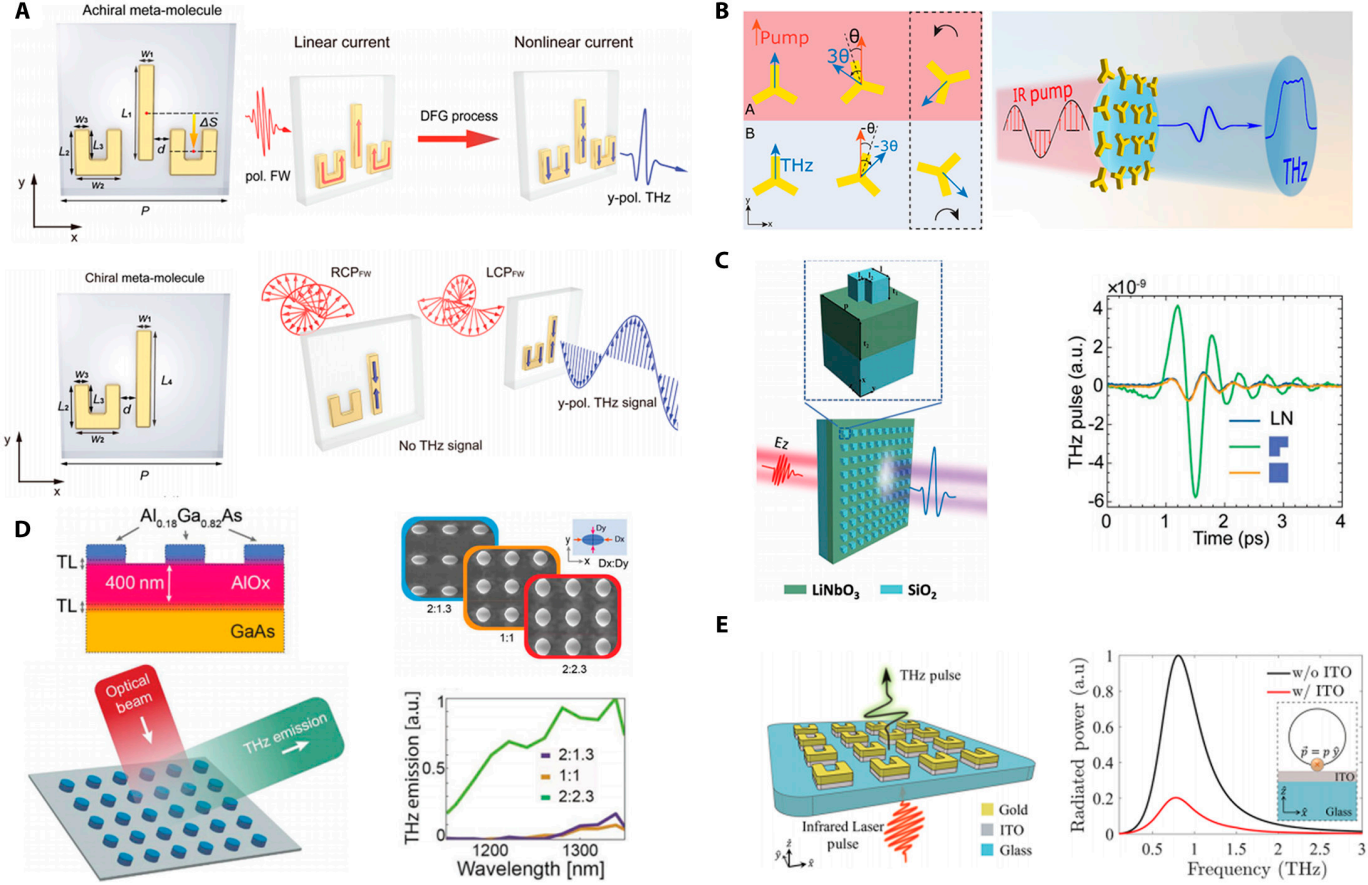
THz generation and manipulation from MTEs. (A) THz generation and manipulation from plasmonic MTEs composed of SRRs and bar resonator. (B) THz generation and high-resolution manipulation from a plasmonic MTE composed of C3 nanostructures. (C) THz generation from all-dielectric MTE composed of SiO_2_ nanoresonators on LiNbO_3_ film. (D) THz generation from all-dielectric MTE composed of AlGaAs nanocylinders on AlO*_x_* substrate. (E) THz generation from a hybrid MTE composed of SRRs on etched ITO film. (A) Reprinted with permission from [100]. Copyright 2023 Wiley-VCH. (B) Reprinted with permission from [43]. Copyright 2023 American Chemical Society. (C) Reprinted with permission from [101]. Copyright 2022 Wiley-VCH. (D) Reprinted with permission from [102]. Copyright 2024 Wiley-VCH. (E) Reprinted with permission from [103]. Copyright 2022 American Chemical Society.

Apart from binary manipulation, near-complete amplitude control, polarization selectivity, and complex wavefront shaping of THz waves were recently demonstrated by Wang et al. [100] using an MTE that employs coupling effects between meta-atoms (Fig. 2A). The MTE consists of meta-molecules with coupled bright and dark meta-atoms, achieving efficient THz emission via difference frequency generation (DFG) under femtosecond IR laser excitation. Coupling between the electric dipole resonance of the bar resonator and the magnetic dipole resonances in the SRRs enables amplitude control via constructive/destructive interference (achiral case) or chiral selectivity (chiral case). Achiral meta-molecules provide up to 93.8% modulation depth in THz emission amplitude, while chiral meta-molecules enable handedness-selective nonlinear THz responses with near-unity nonlinear circular dichroism (NLCD). This controlled coupling approach allows tuning of both amplitude and phase without altering the meta-atom geometry, enabling highly customizable, integrated THz generators and manipulators.

Besides the SRR structure, the C3 symmetry gold plasmonic structure has also been fully explored for THz manipulation and generation. Li and Ellenbogen’s group [34] demonstrated an MTE composed of rotated C3 meta-atoms. THz emission was achieved through DFG upon NIR pump excitation. A selection rule indicating that the THz emission is suppressed for circularly polarized pump waves but activated for linearly polarized pumps was derived under the scenario. Pancharatnam–Berry (PB) phase imposed by the C3 meta-atoms further provides precise control over the phase, polarization, and spatial properties of the emitted THz waves through the orientation of the meta-atom and pump light polarization. Subsequently, the group demonstrated THz metagrating emitters that provide full control over THz beam steering and linear polarization based on PB phase manipulation [105]. More recently, the group reported a highly functional nonlinear MTE facilitating precise high-resolution wavefront manipulation of emitted THz radiation [43]. The MTE consists of super-cells, each containing 2 adjacent C3 meta-atoms as shown in Fig. 2B. THz emission is generated through OR and influenced by both individual C3 meta-atom orientation and near-field superposition within the super-cells. This near-field interference allows rigorous control over the emitted wavefront in terms of amplitude and phase, achieving an extremely high resolution of 600 pixels per THz wavelength. Simulations demonstrated that using the structured Top-Hat beam generated by the MTE can significantly improve performance of high-resolution imaging compared to conventional Gaussian beams. The highly customizable THz emission enabled by MTE is promising for applications requiring precise THz field control, such as bio-imaging, tomography, and high-resolution metrology.

#### THz generation and manipulation from dielectric metasurfaces

While plasmonic MTEs enhance THz radiation through tailored plasmonic resonances, inherent ohmic losses in metals limit their efficiency. In contrast, all-dielectric metasurfaces, free from intrinsic ohmic loss, support high *Q*-factor resonances, offering superior field confinement for nonlinear optical interactions. Unlike plasmonic MTEs, where surface OR dominates THz generation, all-dielectric MTEs provide access to various physical mechanisms [101,102,106,107]. Their high damage thresholds and resistance to thermal effects make them excellent for handling high-power laser excitation, making all-dielectric MTEs powerful candidates for THz generation and manipulation.

Hale et al. [106] delved into the physical mechanisms behind THz radiation from an ultrathin (160 nm) GaAs all-dielectric MTE. Apart from the shift current mechanism, previously overlooked surface nonlinearity has also been found to contribute to THz emission. Joint effects result in MTE achieving up to 4 times greater THz amplitude compared to unpatterned GaAs of the same thickness, comparable to emission from a 650-μm-thick GaAs crystal. Convenient binary control of radiated THz pulse was demonstrated through an MTE made of InAs crystal [107], belonging to the same symmetry group as GaAs. The InAs metasurface generates THz pulses with opposite polarity compared to a continuous InAs layer of the same thickness. This polarity flip arises due to the dominance of the surface OR mechanism in the metasurface, whereas the uniform InAs layer relies on a combination of the surface and volume OR, and photoexcited current transients. The InAs metasurface demonstrates 3 to 4 times higher THz amplitude than a 1-mm-thick ZnTe crystal, making it one of the most efficient table-top THz sources.

Resonant phenomena, such as quasi-bound states in the continuum (quasi-BIC) and Mie resonance, have also been explored to enhance THz emission in all-dielectric MTEs. By intentionally breaking the mirror symmetry of the metasurface’s geometry using L-shaped SiO₂ nanoresonators arranged in a square lattice atop a lithium niobate (LN) film, quasi-BIC modes are introduced [101] (Fig. 2C). These modes facilitate the coupling between a typically nonradiative magnetic dipole mode and a radiative electric dipole mode. As a result, the quasi-BIC effectively directs a substantial portion of the incident NIR optical energy into the LN film's subwavelength volumes, leading to a remarkable 17-fold increase in THz emission amplitude across a broad bandwidth compared to a bare LN film. More recently, Peters et al. [102] demonstrated an all-dielectric MTE consisting of AlGaAs nanocylinders over a 400-nm-thick AlO*_x_* substrate (Fig. 2D). By tailoring the geometry of the nanocylinders to support Mie resonances, the internal optical fields are significantly enhanced, leading to a 40-fold increase in THz emission efficiency compared to the bare substrate. This design allows for control over the phase and amplitude of the emitted THz radiation by adjusting the nanocylinder shapes and pump wavelengths, enabling potential complex wavefront manipulation.

#### THz generation and manipulation from hybrid metasurfaces

To further amplify the nonlinear processes at metal interfaces, hybrid nonlinear MTEs have been explored. Materials with epsilon-near-zero (ENZ) properties have emerged as compelling alternatives for enhancing nonlinear interactions [108–111]. These materials exploit the substantial field intensification resulting from the continuity of the normal displacement field at the ENZ boundary [103,111–113]. Incorporating ENZ films with metasurfaces can significantly strengthen the nonlinear response by utilizing the concentrated near-field effects of resonant meta-atoms at target frequencies.

Lu et al. [108] reported a nonlinear MTE composed of gold SRRs on top of a 23-nm-thick indium tin oxide (ITO) film. The coupling between the SRR’s plasmonic resonance and the ENZ mode of the ITO film can enhance the THz emission by more than 4 orders of magnitude spanning over 2 THz. Angle-resolved circularly polarized (CP) THz wave and CP-dependent vortex have been easily achieved by rotating the SRRs. The ITO film underneath the metasurfaces plays a critical role in enhancing the local electric field at its ENZ region, significantly boosting the nonlinearity of the system. However, ITO’s metallic behavior in THz regime lowers radiation efficiency, damping free-space THz emission from metasurface. To address this issue, Ellenbogen’s group [103] etched away the ITO layer surrounding meta-atoms while preserving ITO underneath SRRs as shown in Fig. 2E. This fabrication approach maintains local field enhancement from ITO’s ENZ response while preventing THz radiation damping. The etched metasurface exhibited a 14-fold increase in radiated power compared to unetched samples.

### Hybrid systems

**Fig. 3.**
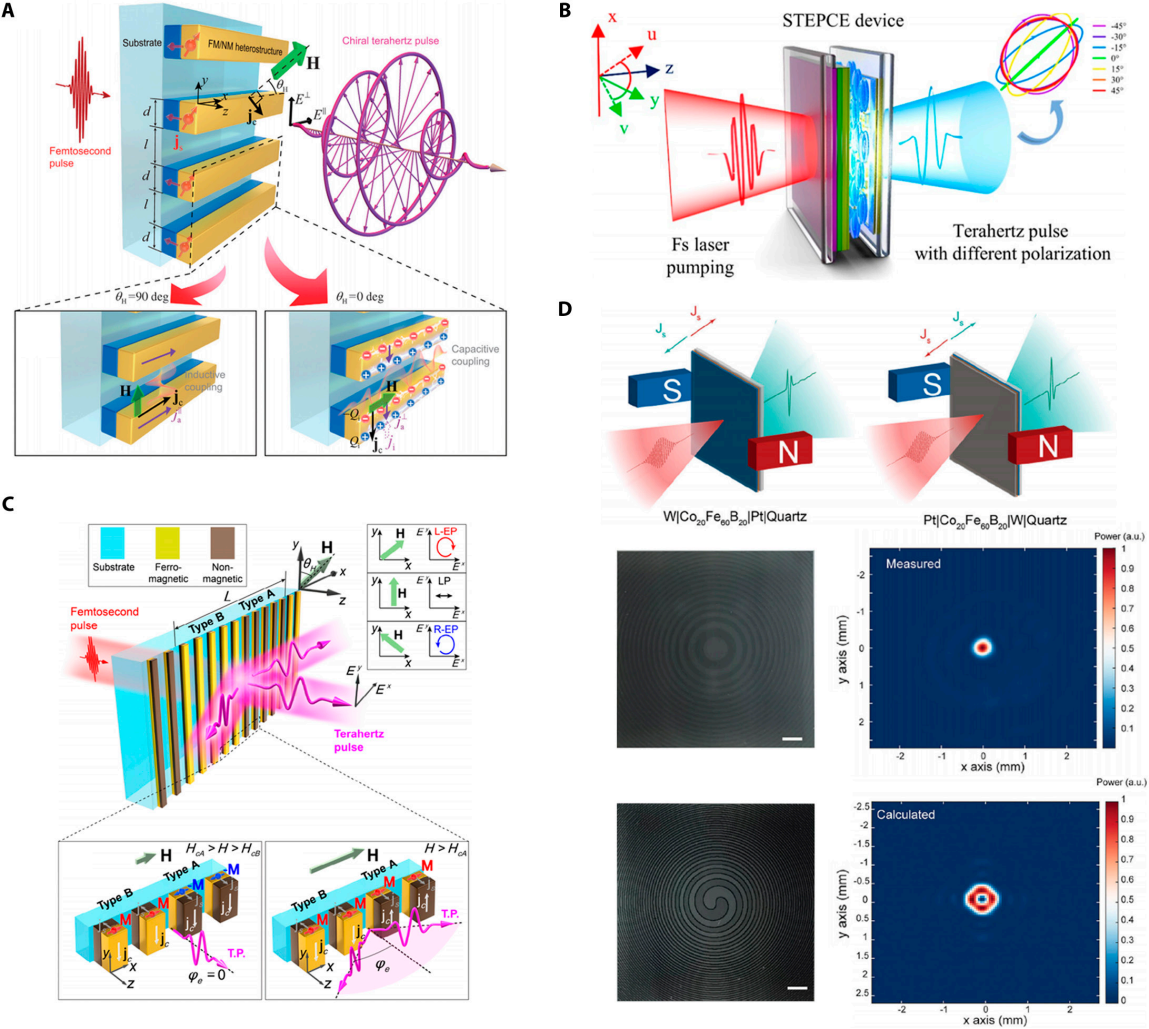
THz generation and manipulation from hybrid systems. (A) Generation and polarization control from a spintronic-metasurface terahertz emitter (SMTE). (B) Generation and polarization control through a hybrid system containing a heterostructure, liquid crystal, and a metasurface. (C) Generation, polarization control, and beem steering from an SMTE. (D) Generation and wavefront modulation from an SMTE. (A) Reprinted with permission from [31]. Copyright 2021 SPIE and Chinese Laser Press. (B) Reprinted with permission from [44]. Copyright 2022 American Chemical Society. (C) Reprinted with permission from [32]. Copyright 2022 American Chemical Society. (D) Reprinted with permission from [115]. Copyright 2024 Wiley-VCH.

Apart from the aforementioned attractive features of heterostructure-based STEs, the STEs are highly suitable for integration with other materials or structures, offering new possibilities for STE device development [31,44,74,114]. Recently, inspired by metasurfaces’ ability to arbitrarily tailor the electromagnetic (EM) wave responses, hybrid systems have been explored for simultaneous generation and flexible manipulation of THz radiation. Joint effects of ISHE and capacitive coupling induced by stripe patterns on a spintronic-metasurface THz emitter (SMTE) enable efficient, flexible generation of broadband (1 to 5 THz) chiral THz waveforms, with ellipticity over 0.75 [31] (Fig. 3A). A hybrid system composed of a W/CoFeB/Pt trilayer heterostructure, liquid crystal, and an SRR metasurface is reported to achieve chiral THz waveforms with ellipticity higher than 0.9 [44] (Fig. 3B).

To address the increasing demands in THz technology applications, it is crucial to explore more integrated, compact, and efficient THz sources enabling complex and flexible control directly at the generation stage. The stripe-patterned SMTE has been widely explored for THz generation and wavefront manipulation, as it enables the control of 2 distinct states (“0” and “1”) in a simple and convenient manner, allowing for the realization of a range of complex functionalities. Tong et al. [41] used Co/Pt and Co/W heterostructures with different THz radiation behaviors due to Pt and W’s opposite spin Hall angles to achieve binary control of the generated THz waves. A grating-like Ge film is applied to achieve real-time manipulation of the radiated THz field. Wang et al. [32] proposed a multi-functional SMTE, which inherits the advantages of both metasurface and standard STEs. The SMTE consists of 2 types of heterostructures: FM (Fe, 1.5 nm)/NM (Pt, 3 nm) (type A) and NM (Pt, 3 nm)/FM (Fe, 1.5 nm) (type B). Type A structure includes an AFM layer [CoO (3 nm)/NiO (1.5 nm)] to enhance coercive field strength via exchange interaction. A 4-nm-thick Al_2_O_3_ layer in type B balances thickness difference. These heterostructure-based meta-atoms are periodically stacked, forming a metasurface lattice of stripes as illustrated in Fig. 3C. By controlling the external magnetic field, magnetization in different stripes can be tuned, allowing beam steering through constructive and destructive interference based on the metagrating arrangement. Additionally, the THz polarization can be independently controlled by manipulating the magnetic field angle, enabling full polarization adjustment across a wide range of emission angles. Reversing the typical W/CoFeB/Pt trilayer heterostructure to Pt/CoFeB/W also enables the convenient binary control and manipulation of THz emission and wavefront. Chen et al. [115] reported the generation of THz dual-beam, THz FZP, and THz vortex beam by utilizing reversed trilayer configurations (W/CoFeB/Pt and Pt/CoFeB/W), as shown in Fig. 3D.

## Modulation

THz wave modulation is crucial for application in communication, beam shaping, sensing, and imaging [13,116,117]. Although few reported SMTEs achieved simultaneous generation and modulation of the emitted THz wave, the modulation mechanism is always passive, meaning once the device is fabricated, the functions are fixed. Traditional THz modulators often struggle with limited flexibility and efficiency. However, advancements in nanoengineered platforms, especially metasurfaces, have opened new avenues for dynamic and precise control over THz waves during the past decade [11,117]. By integrating active materials such as graphene [58,118–121], semiconductors [55,57,122–127], liquid crystals [6,14,53,54], perovskites [128–130], superconductors [131,132], magneto-optic materials [59,133], phase-change materials [56,134–148], high electron mobility transistors (HEMTs) [149–151], and micro-electromechanical systems [152–154] metasurfaces, researchers have achieved unprecedented active modulation of amplitude, phase, and polarization. These innovations not only enhance the performance of THz modulators but also enable real-time reconfigurability and independent control of individual elements, paving the way for sophisticated THz spatial light modulators. This section delves into the state-of-the-art developments in THz modulation, highlighting the transformative impact of nanoengineered solutions on the manipulation of THz waves**.**

### Homogeneous THz modulators

Homogenous THz modulators have been extensively studied through the integration of periodic metallic or dielectric structures with active materials, where the amplitude, phase, and polarization of THz wave in each unit cells exhibit uniform variation in response to external stimuli. The amplitude, phase, and polarization of THz wave can be designed by exploiting the resonant responses of metasurfaces, which are influenced by the refractive index or conductivity of the surrounding materials. By incorporating THz metasurfaces with active materials, it becomes possible to dynamically adjust the amplitude, phase, and polarization of THz wave by altering the refractive index or conductivity of active materials. THz modulators can be categorized based on their modulation objectives, which include amplitude modulation, phase modulation, and polarization modulation.

#### Amplitude modulation

**Fig. 4.**
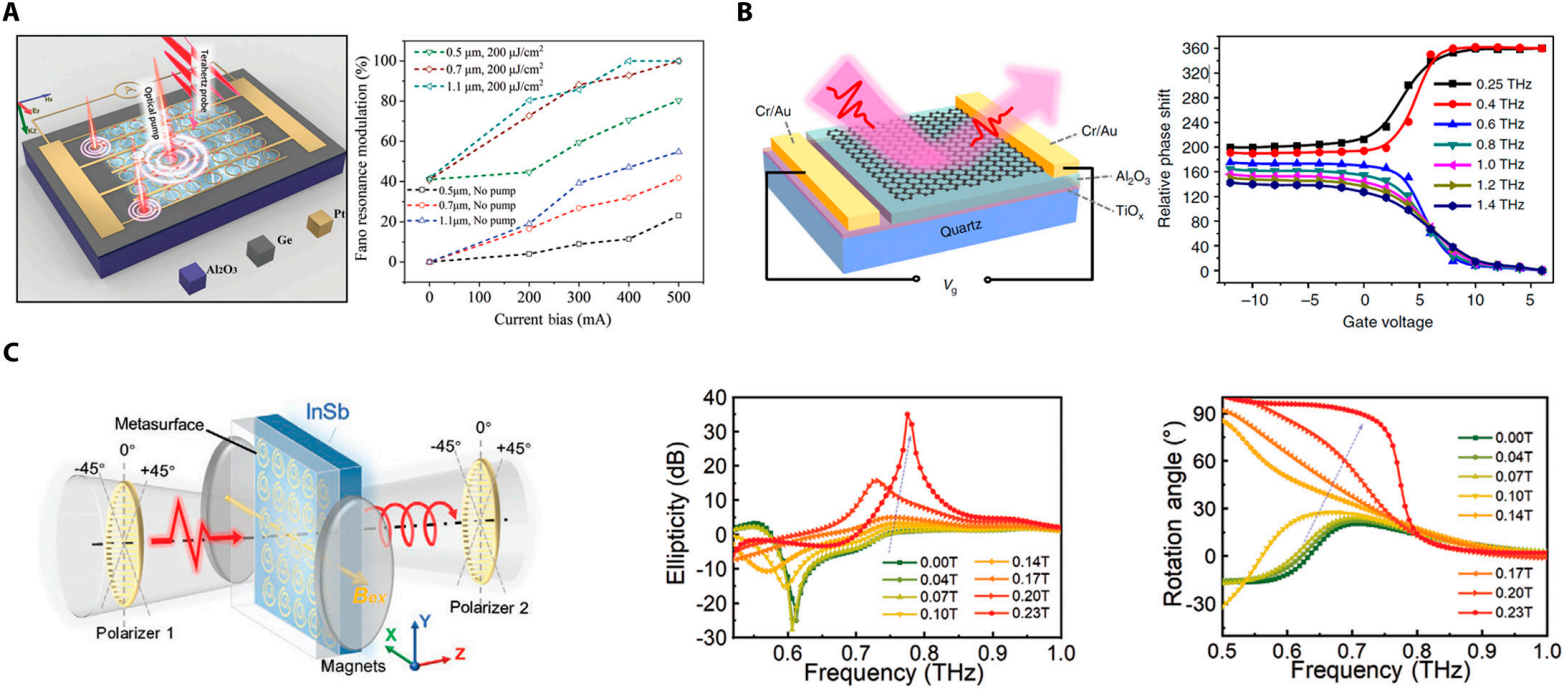
Homogeneous THz modulators. (A) Ultrafast dual-stimulus THz amplitude modulator by integrating asymmetric SRRs with Ge thin film. (B) Utrabroadband THz phase modulator based on the graphene-controlled Brewster angle device. (C) THz polarization modulator based on the magneto-optical metasurface. (A) Reprinted with permission from [55]. Copyright 2021 Wiley-VCH. (B) Reprinted with permission from [58]. Copyright 2018 Springer Nature. (C) Reprinted with permission from [59]. Copyright 2021 Wiley-VCH.

THz amplitude modulators play a pivotal role in a range of applications, including communication, imaging, and spectroscopy. The principle parameters that characterize amplitude modulation are modulation depth, modulation bandwidth, and modulation speed. The dynamic tuning of amplitude can be achieved by switching or shifting the resonance of THz metasurfaces through the use of active materials. For example, Cai et al. [56] demonstrated the electrically and optically tunable THz metadevices by integrating VO_2_ patterns with asymmetric SRRs. Under electrical stimulation, they achieved an absolute modulation depth of up to 54% with a switching time of 2.2 s. Furthermore, ultrafast modulation within 30 ps can be achieved by exploiting the femtosecond pulse-induced metal-insulator transition of VO_2_. In contrast to the volatile nature of VO_2_, the phase transition of Ge_2_Sb_2_Te_5_ (GST) is nonvolatile and can be sustained at room temperature. Liu et al. [146] reported a nonvolatile reconfigurable electromagnetically induced transparency (EIT) by incorporating GST into the meta-atoms. A significant change in conductivity can be induced by the reversible amorphous-crystalline phase transition of GST. Under the excitation of nanosecond laser pulses, the EIT resonance can be reversibly switched, yielding a modulation of 56% at 0.92 THz. Unlike most THz modulators that respond solely to a single external driving field, Lou et al. [55] proposed an ultrafast dual-stimulus THz metadevice by integrating asymmetric SRRs with a Ge thin film, as shown in Fig. 4A. An 800-nm optical pump of 1,600 μJ/cm^2^ enables a modulation depth of 100% for Fano resonance, with an ultrafast switching time of less than 10 ps. By introducing current bias, the electro-induced carriers facilitate the photo-generated carriers in lowering the threshold for pump fluence to 200 μJ/cm^2^. In comparison with the single optical stimulus, the THz amplitude modulation is enhanced by 56.3% under dual-stimulus control.

#### Phase modulation

Phase control is important for applications such as beam shaping, beam steering, and holographic imaging [155]. The phase of the emitted EM wave undergoes a large variation around the resonant frequency of metasurfaces. Therefore, THz phase modulation can be achieved through the dynamic tunability of the resonant frequency using active materials. For instance, Nouman et al. [138] proposed a method for phase and polarization modulation of THz waves by integrating a hybrid metasurface with VO_2_ patches. By altering the input bias current from 100 to 300 mA, the resonant frequency can be tuned from 0.52 to 0.37 THz, resulting in a phase modulation of 64° within the frequency range of 0.45 to 0.47 THz. Additionally, the phase difference between orthogonally polarized incident fields varies from 78 to 14°, allowing for the transmitted polarization to switch from approximately circular to linear. Zhang et al. [150] achieved a large phase modulation of THz waves by incorporating a resonant metamaterial with a nanostructured two-dimensional electron gas (2DEG) layer of a GaN HEMT. By electrically tuning the carrier distribution and depletion of the 2DEG, they were able to control the conversion between 2 distinct coupling modes, resulting in a significant phase modulation of THz waves. A transmission phase shift of 137° was achieved at 0.35 THz with the application of a voltage ranging from 0 to 8V. However, the THz modulators based on active resonant metasurfaces typically exhibit a narrow bandwidth. In contrast, Chen et al. [58] demonstrated a graphene/quartz THz modulator characterized by near-perfect tunability, ultrabroad operation bandwidth, and rapid modulation speed, as illustrated in Fig. 4B. The Brewster angle can be tuned from 65° to 71° by varying the conductivity of graphene. By appropriately selecting the incident angle, the device can function as a THz-intensity modulator with a spectrally flat modulation depth of 99.3 to 99.9% across a wide frequency range from 0.5 to 1.6 THz while also serving as a phase modulator with a tunability exceeding 140°. In recent years, liquid crystals are also employed to integrate with metasurfaces to realize THz phase modulation and wavefront control [6,14,53,54,156–158]. For instance, Wu et al. [54] realized a relative phase difference of π with the same reflection amplitude at 0.67 THz by applying electric field to adjust the orientation of liquid crystals. Moreover, dynamic beam steering was demonstrated by electrically addressing each element independently. Additionally, Zhao et al. [158] proposed a THz liquid crystal cascaded metadevice, including a liquid crystal layer, an asymmetric metasurface layer, and a helical phase metasurface layer. Dynamic conversions between ±2-order vortex beam and vector beam were achieved by electrically controlling the orientations of liquid crystals.

#### Polarization modulation

Metasurfaces provide an excellent platform for manipulating and detecting the polarization state of THz waves. Recently, the polarization states of incident THz waves are determined by utilizing all-silicon metasurfaces based on the polarization multiplex techniques [159,160]. Moreover, off-axis vector vortex beams with arbitrary inhomogeneous polarization states can be generated by employing all-silicon metasurfaces [161]. The dynamic control of THz polarization is crucial for the development of beam splitters, isolators, and polarimetric imaging. By combining anisotropic metasurfaces with active materials, the THz polarization can be dynamically tuned under external stimulus. For example, Wang et al. [53] demonstrated a tunable THz waveplate by utilizing liquid crystals in conjunction with porous graphene and subwavelength metal wire grid. The birefringence of the liquid crystals allows for the modulation of phase retardation between 2 orthogonal components of the THz wave by adjusting the applied voltages. At a frequency of 2.1 THz, the output polarization states transitioned from a linear polarization (half-wave plate) to elliptical, then circular (quarter-wave plate), and again elliptical polarizations, and ultimately back to an orthogonal linear polarization as the voltage increased. Fan et al. [59] proposed a dynamic THz anisotropy and chirality by utilizing a magneto-optical metasurface composed of an asymmetric metallic metasurface on the InSb substrate, as presented in Fig. 4C. The gyroelectric semiconductor InSb exhibits strong THz magneto-optical effects. As the external magnetic field is intensified, the polarization state at 0.7 THz rotates from 45° to 90°, while the output wave at 0.8 THz transitions from a linear polarization to a right-circular polarization. Moreover, the conversion rate from right-circular polarization to left-circular polarization increases dramatically by nearly 100% within the 0.6- to 0.75-THz frequency range, whereas the conversion rate from left-circular polarization to right-circular polarization diminishes to nearly 0 in the same band. Additionally, a magnetic Weyl semimetal metasurface was proposed by combining a geometric phased metasurface with InSb [162]. Flexible THz beam steerings with 4 different working modes were demonstrated by changing the direction of the magnetic field. Besides the InSb, rare-Earth-doped yttrium iron garnet (YIG), as another magneto-optical material, is combined with an anisotropic metasurface and a Pancharatnam–Berry metasurface to generate a THz magneto-optical spin-modulated metadevice [163]. Spin-selective beam steering is dynamically manipulated by external magnetic field, and power distribution can be tuned with the max modulation depth of 91.6%. Recently, Hu et al. [127] proposed a broadband THz polarization modulation operating on a picosecond timescale, utilizing an achromatic Fano metasurface combined with a thin amorphous Ge layer. This configuration facilitates uniform broadband cross-polarized transmission, achieving an average amplitude of 0.32 across the frequency range of 0.6 to 1.1 THz. Notably, the cross-polarized transmission exhibits significant suppression as the pump fluence increases to 1.2 mJ/cm^2^. The modulation depth can reach up to 90%, with an on–off–on switching cycle of less than 10 ps.

### Spatial light modulators

While substantial advancements have been made in the development of THz modulators utilizing active THz metasurfaces, these devices generally modulate the amplitude, phase, and polarization of THz waves uniformly across the entire structure, rather than allowing for independent control of each individual element. To facilitate more versatile and comprehensive manipulation of THz waves, it is essential for the intensity, phase, and polarization of each discrete element to be reconfigurable in real time, enabling the creation of THz spatial light modulators. Recent studies have reported the emergence of both amplitude- and phase-type THz spatial light modulators, achieved through the independent electrical or optical tuning of the amplitude or phase of individual elements [6,14,54,57,126,141,148,151].

#### Phase-type spatial light modulators

**Fig. 5.**
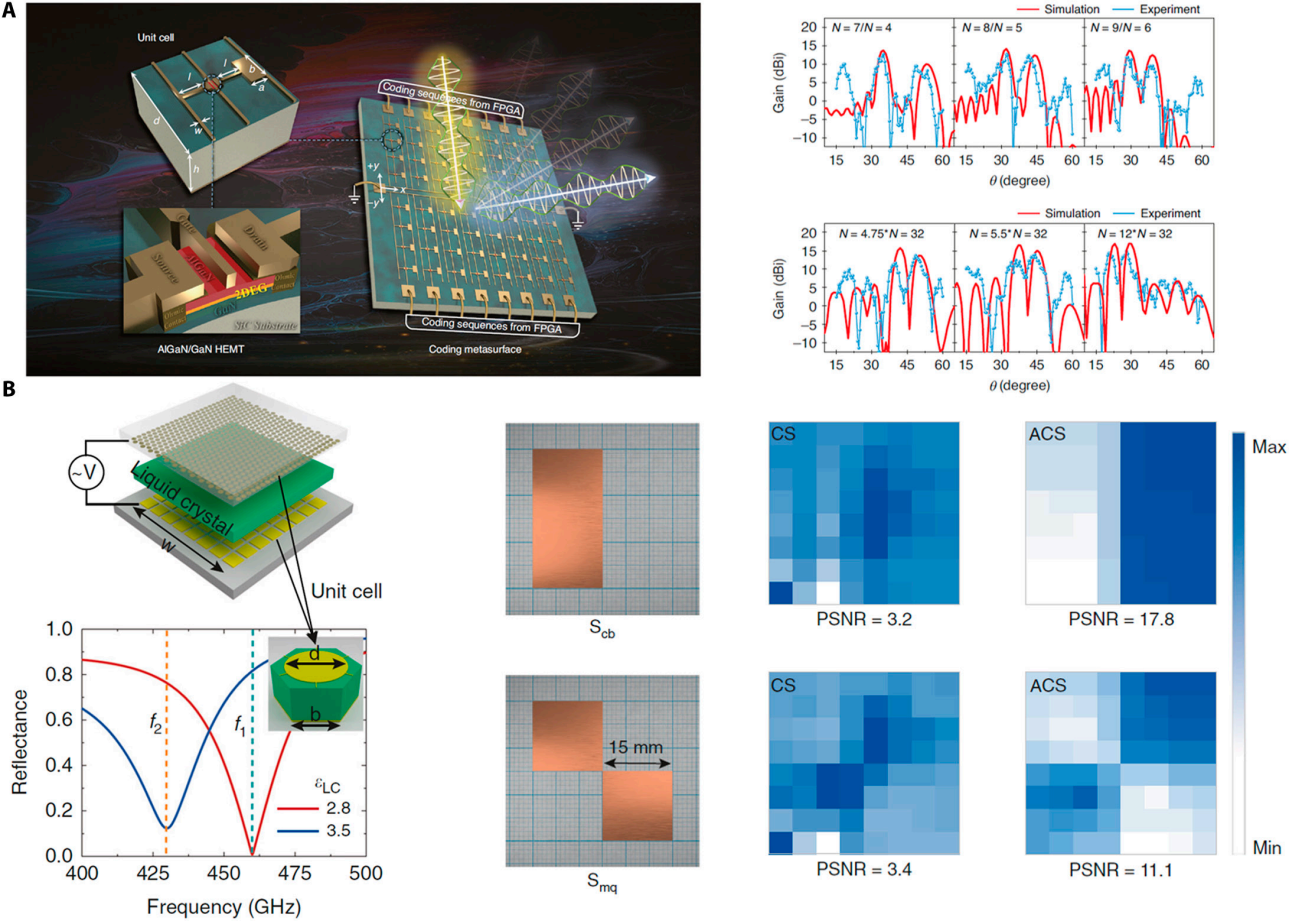
THz spatial light modulators. (A) Phase-type THz spatial light modulators by utilizing a real-time controllable THz programmable metasurface. (B) Amplitude-type THz spatial light modulators based on the tunable liquid crystal metasurface absorber. (A) Reprinted with permission from [151]. Copyright 2023 Springer Nature. (B) Reprinted with permission from [14]. Copyright 2022 Springer Nature.

Phase-type THz spatial light modulators facilitate the precise manipulation of wavefronts, presenting significant potential applications in THz wireless communication, sensing, and imaging. The phase of each unit cell must be independently modulated while maintaining a constant amplitude. For instance, Venkatesh et al. [126] showcased large-scale programmable THz metasurfaces through the use of arrays composed of complementary metal-oxide semiconductor (CMOS)-based chip tiles, as shown in Fig. 5A. Each element within these arrays is individually addressable and can be digitally programmable with 8-bit control at GHz speed. The amplitude modulation at 0.3 THz achieves a level of 25 dB, with a phase variation of approximately 260°. Furthermore, dynamic beamforming across an angular range of ±30° is accomplished by engineering various phase gradients. Additionally, the implementation of binary-amplitude-only holography is achieved by switching the digital states of the unit cells. The integrated chip operates at a voltage of 1.2 V, exhibiting a static power consumption of 240 μW. Recently, Lan et al. [151] reported a real-time controllable THz programmable metasurface that incorporates 2DEG into the asymmetric resonant structures. By applying a bias to modulate the carrier concentration in the 2DEG, the phase of the reflected wave at 0.34 THz can be switched by 180° while maintaining a uniform amplitude, corresponding to the ideal “on” and “off” states in a 1-bit coding scheme. This system has achieved wide beam scanning with an accuracy of 1°. In addition, their work has demonstrated capabilities for multi-beam generation, diffuse scattering, and point-to-point signal transmission. The HEMT offers several advantages, including a substantial dynamic range of carrier density, a high electron drift velocity, reduced parasitic capacitance, and lower power dissipation. Besides the independently electrical control, Guo et al. [57] proposed a phase-type THz spatial light modulator utilizing an all-optical dynamically reconfigurable metasurface. This metasurface is formed by illuminating a thin silicon wafer with a femtosecond laser pump beam, which is modulated by a digital micromirror device (DMD). The silicon regions exposed to illumination become conductive due to the generation of photocarriers, which serve as the subwavelength resonators of THz metasurfaces. The arbitrary phase profile of the THz beam can be generated by controlling the orientation of individual resonators using the DMD. Demonstration of dynamic holographic imaging and reconfigurable zoom lens have been achieved by reprogramming the DMD to create various patterns of pump beam illumination.

#### Amplitude-type spatial light modulators

In addition to independently controlling the phase of each unit cell, the amplitude of each unit cell can also be individually manipulated. For example, Li et al. [14] presented an electrically programmable dual-color THz amplitude-type spatial light modulator that utilizes a tunable liquid crystal metasurface absorber, as illustrated in Fig. 5B. The liquid crystal layer is sandwiched between a metallic metasurface and a metallic ground layer, allowing for electrical control of each pixel via a field-programmable gate array (FPGA). The amplitude modulation depth of the tunable metasurface absorber can reach higher than 70% at 2 distinct frequencies. Furthermore, dual-color compressive sensing (CS) imaging for dispersive objects is achieved by leveraging the significant frequency shift that can be controlled by an external electric field. In previous demonstrations of THz spatial light modulators, the application of external voltage or optical pump was necessary to sustain modulation functions, resulting in elevated energy consumption. Recently, Chen et al. [148] introduced a lithography-free photo-imprint technique for the development of reconfigurable and nonvolatile THz elements based on the phase-change material GST. Amorphous and crystalline GST exhibit a large transmission contrast in the THz band. They employed a single patterned pump pulse to spatially modulate the re-amorphization of crystalline GST, leading to a high-contrast pattern of the amorphous state on the GST film. Through careful design of the pump pattern, a series of reconfigurable and nonvolatile ultrathin (100 nm) THz flat lenses with subwavelength-scale and ultra-broadband focusing performance were experimentally demonstrated.

## Bio-Applications

In addition to the prosperous and thriving advancement in nanoengineered THz generation and modulation, exciting progress on biosensing and biofunction techniques have been made in the THz region. Molecular resonant absorption accompanies with vibration fingerprints [9,18,67,164], including biological molecules, organic molecules, van der Waals forces, and hydrogen bonding, rendering it a suitable technology to achieve label-free biosensing detection and biomolecule function adjustments. Such phenomena often happen in the THz region and extend to the IR. In this section, we would focus on the intersection between physics and biology, and describe in detail the related applications for detection of bioanalytes and the regulation of biofunctions based on THz photonics.

### Biosensing techniques

Although conventional analytical methods such as labeled immunoassays, polymerase chain reaction, cell culturing, and light microscopy still lay the foundation of modern clinical detection systems [165,166], high-sensitivity, low-cost, easy-to-use, and point-of-care biosensors would be a game-changer for the future management of diseases, which can be applied in virtually any location [167]. However, the improvement of detection sensitivity is seriously hampered due to the limited light–matter interactions and the scale mismatch between THz waves and bioanalytes. Recent advancements in metamaterials and metasurfaces [11,42] enable flexible engineering of arrangement patterns and resonances to enhance sensing capabilities. These technologies concentrate electromagnetic energy into subwavelength volumes, creating hotspots with strong electric fields. By adjusting structural parameters such as materials, geometry, and arrangements, metasurface sensors can be designed to exhibit varied configurations and resonance mechanisms like Fano, toroidal, and BICs to enhance the light–matter interaction [168–170]. Unlike other reviews focusing on metasurface design [10,12,60,77], we provide a comprehensive introduction to biosensors for detecting refractive index changes and characterizing molecular fingerprints.

#### Sensors based on refractive index change

Discarding the fingerprint spectrum, researchers typically cover analytes on the metasurface sensor to obtain specific dielectric information according to the effective medium theory. The resonance frequency shift and amplitude modulation result from the refractive index and extinction coefficients, respectively. For better acquiring sensitive and reliable sensing data, researchers have dedicated significant effort to refractometric affinity biosensing, aiming to improve sensitivity, specificity, and accuracy.

**Fig. 6.**
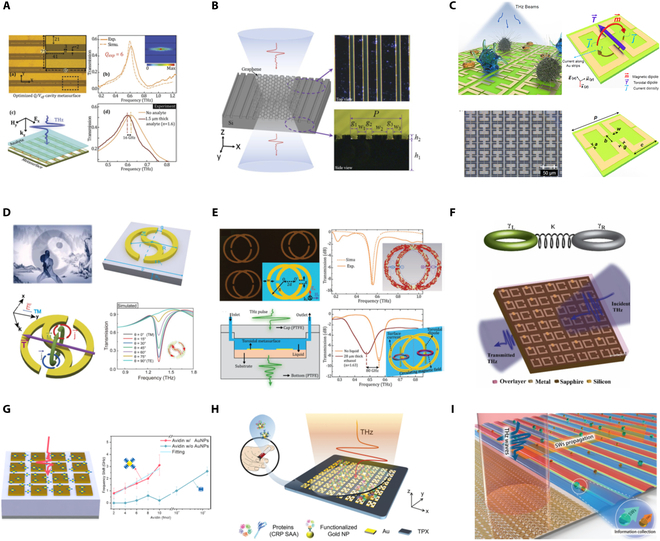
Improvement of refractive sensitivity. (A) THz metasurface cavities with sensitivity of 61 GHz/RIU. (B) Ultrasensitive THz metasurface based on silicon Fano resonance. (C) THz toroidal resonance for sensitive distinction of lung cancer cells. (D) High-sensitivity toroidal metasurface for concentration detection of Aβ protein. (E) THz microfluidic biosensing with dual-toroidal metasurfaces. (F) Exceptional point (EP) metasurface achieving a maximum sensitivity of up to 0.063 THz/RIU. (G) AuNP-based metasurface sensing with 1,000-fold sensitivity improvement. (H) AuNP-assisted BIC metasurfaces for detecting ultralow-concentration analytes. (I) High-sensitive THz refractive sensing through metasurface-excited SWs. (A) Reprinted with permission from [171]. Copyright 2020 Wiley-VCH. (B) Reprinted with permission from [172]. Copyright 2021 IEEE. (C) Reprinted with permission from [173]. Copyright 2021 De Gruyter. (D) Reprinted with permission from [61]. Copyright 2023 De Gruyter. (E) Reprinted with permission from [174]. Copyright 2021 Wiley-VCH. (F) Reprinted with permission from [175]. Copyright 2024 IOP Publishing. (G) Reprinted with permission from [176]. Copyright 2016 American Chemical Society. (H) Reprinted with permission from [177]. Copyright 2023 Wiley-VCH. (I) Reprinted with permission from [178]. Copyright 2024 Wiley-VCH.

Refractive sensitivity is defined as the wavelength shift in the output spectrum relative to the refractive index change, with factors surface sensitivity (S), figure of merit (FOM), and resonance quality (Q) often considered [164]. Various resonance mechanisms have been explored and refined using metallic and dielectric materials. For instance, Gupta and Singh [171] excited high *Q* resonances with effectively low mode volume, which can achieve sensitivity levels of 61 GHz/RIU (refractive index unit) experimentally, as shown in Fig. 6A. Zhong et al. [172] presented an ultrasensitive THz metasurface based on silicon Fano resonance to provide strong near-field enhancement and boost light–analyte interaction. A *Q* factor of 39,587 and an FOM of 533 are separately reached, which have significant advantages in the application of refractive index sensing (Fig. 6B). Unlike electric and magnetic dipolar resonance, toroidal metasurface originating from the alignment of magnetic moments has the ability to excite sharp resonance and has been widely used in the biosensing realms [19]. It is reported that a novel label-free and low-cost strategy is proposed for rapid detection and distinction of lung cancer cells based on THz toroidal metasurfaces with 485.3 GHz/RIU [173], as shown in Fig. 6C. Besides, toroidal resonance has been successfully applied to the low-concentration detection (ranging from 0.0001 to 10 mg/ml) of Aβ protein associated with Alzheimer’s disease (Fig. 6D) [61]. Furthermore, by combining with microfluidic technology, dual-toroidal metasurfaces have been designed to enhance sensitivities of polar liquid analytes based on spectral shift and resonance linewidth variation. A value of 124.3 GHz/RIU has been realized for 28-μm-thick microfluidic layers of different mixed ethanol–water solutions (Fig. 6E) [174]. Recently, singularities of non-Hermitian systems, known as exceptional points (Eps), have been realized in a multilayered periodic plasmonic structure to enhance sensitivity [179,180]. KN and Chowdhury [175] obtained the eigen resonance frequencies and loss rates of Ep metasurface to achieve a maximum sensitivity of up to 0.063 THz/RIU, as shown in Fig. 6F. To further improve the sensitivity, some pioneering works have proven that nanomaterials can greatly boost the metasurface performance [176,177]. For example, gold nanoparticles (AuNPs) of 7.8 fmol have been introduced to traditional metasurface to enable a 1,000-fold sensitivity improvement compared with that of avidin alone [176], as shown in Fig. 6G. Besides, Wang et al. [177] presented plasmonic BIC metasurface to detect low-concentration analytes with the help of AuNPs (Fig. 6H). The metasurface sensitivity slope is up to 674 GHz/RIU, enabling the detection of picomolar-level bioanalytes. Traditional metasurface sensors typically rely on a single resonant mode, which is challenging to integrate sensing performance for both the real part of the refractive index and the imaginary part. To address the above bottlenecks, Zhang et al. [178] proposed a new strategy for THz sensing based on surface waves (SWs), as shown in Fig. 6I. By leveraging the superior properties of SWs, highly sensitive refractive sensing and fingerprint spectrum recognition are achieved simultaneously. The proposed sensing strategy not only enables refractive sensing up to 215.5°/RIU but also resolves multiple fingerprint information within a continuous spectrum. This work achieves the integration of highly sensitive sensing in both *n* and *k* for the first time, while being 3 or 4 times more sensitive than conventional sensors. In addition, THz chiral polarization sensing enhanced by chiral metasurfaces has attracted extensive attention to distinguish bio-enantiomers recently [181–183]. For example, Fan et al. [181] investigated a chiral metasurface sensor filled with FM nanofluids to detect magnetic nanoparticles, with sensitivity of 5.5 GHz %^−1^. Shi et al. [182] designed an anapole metasurface sensor to realize the chiral recognition of the amino acid enantiomers, where the highest detection sensitivity is 0.516 GHz•ml/μmol.

**Fig. 7.**
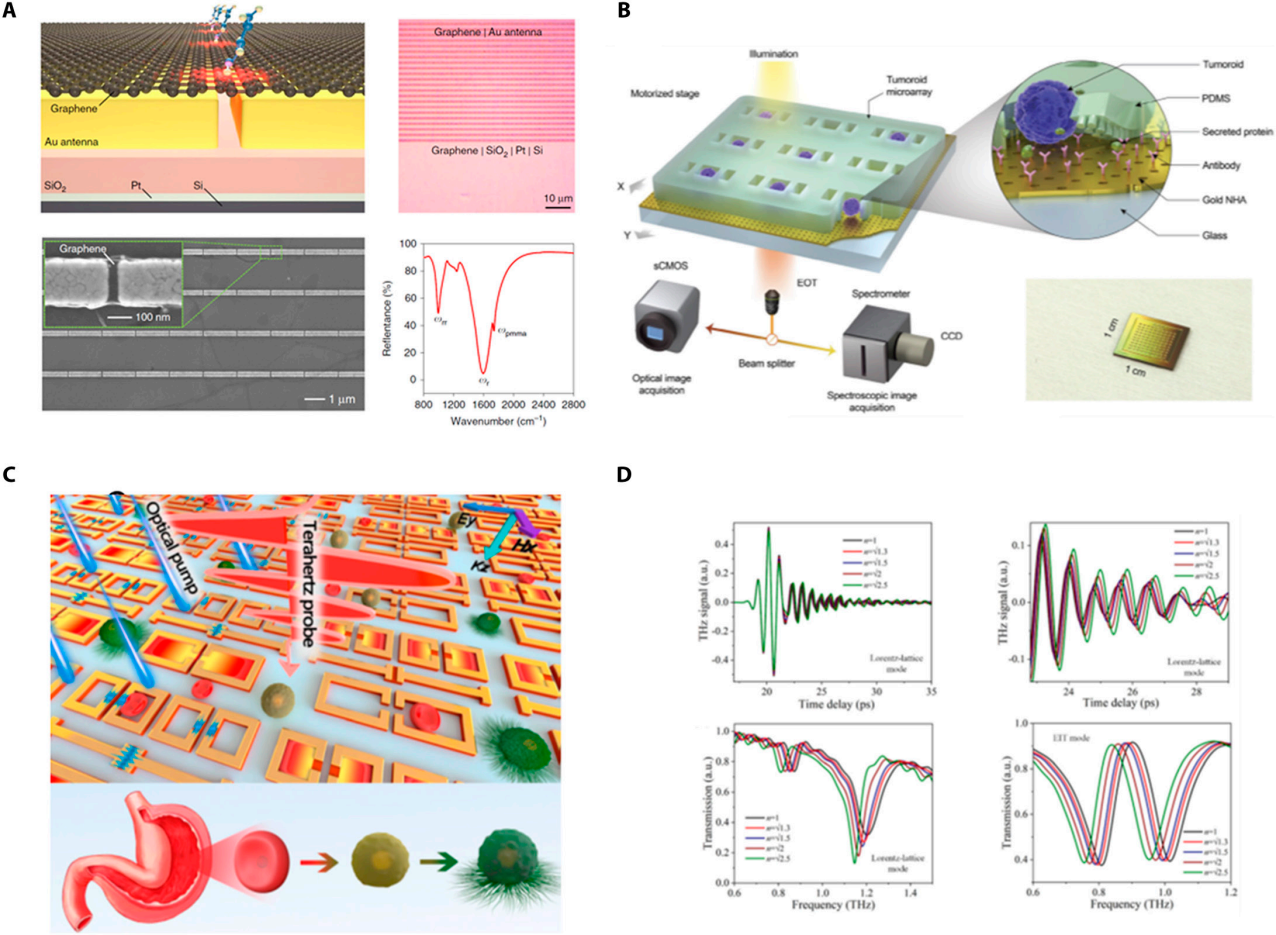
Improvement of refractive specifity and accuracy. (A) Graphene enables the mid-IR metasurface to qualitatively identify glucose. (B) Antibody enables silicon-based metasurfaces to qualitatively identify rabbit anti-mouse IgG. (C) Calibration-free, high-precision, and robust metasurface sensor for monitoring gastric cancers. (D) Two-dimensional THz metasurface sensor with improved accuracy. (A) Reprinted with permission from [22]. Copyright 2018 Springer Nature. (B) Reprinted with permission from [184]. Copyright 2024 Wiley-VCH. (C) Reprinted with permission from [185]. Copyright 2022 National Academy of Sciences. (D) Reprinted with permission from [186]. Copyright 2022 Royal Society of Chemistry.

Considering that bare refractive sensing metasurface inherently lacks analyte specificity, extra surface modification is necessary to equip the devices with bioanalyte selectivity. A series of functional materials have been modified onto the metasurface, such as graphene, antibody, and hydrogel [5,21,22,184,187–190]. For instance, Zhu et al. [22] combined graphene and 45-THz metasurface to achieve highly sensitive detection of low-molecular-weight analytes based on the increase in the graphene optical conductivity with boronic acid pyrene, as illustrated in Fig. 7A. The affinity binding-based quantitative detection of glucose can be down to 200 pM (36 pg/ml). As shown in Fig. 7B, Liu et al. [184] combined optofluidic nanoplasmonic biosensor and label-free spectroscopic imaging to construct detection platform that enables real-time secretion analysis from single tumoroids. The antibodies are pre-functionalized on the sensor surface to specifically detect the secreted protein.

The primary premise to develop practical sensors is to guarantee the accuracy of sensing performance, which requires eliminating measurement errors and optimizing the signal-to-noise ratio as perfectly as possible [185,186,191]. Lou et al. [185] proposed and experimentally demonstrated a calibration-free THz sensor for achieving high-precision biosensing detections, by normalizing the Fourier-transformed transmission spectra between picosecond order time delay of an optically controlled ultrafast metasurface (Fig. 7C). A theoretical framework is established to clarify the unique advantages of the calibration-free strategy on elimination of measurement errors, which is associated with external factors. As a biosensing validation, the cancerous process of gastric cells has been successfully monitored based on the calibration-free metasurface, which is consistent with the cell-staining experiments. Besides, the time-domain and frequency-domain THz platforms are simultaneously built on a single metasurface by Jiao to serve the sensing target and validate each other to decrease the measurement errors [186], as shown in Fig. 7D.

#### Sensors based on surface-enhanced absorption

**Fig. 8.**
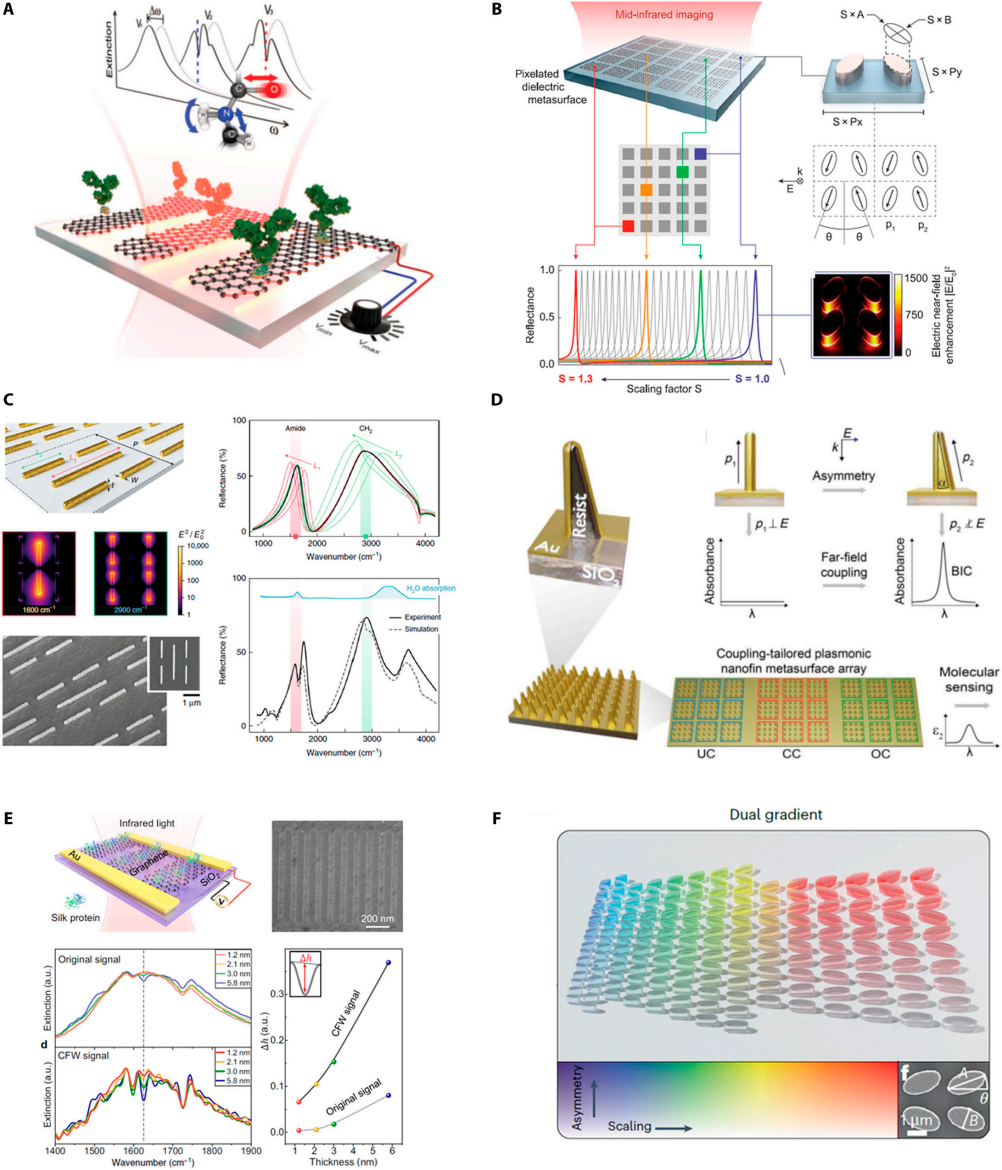
Fingerprint characterization based on SEIRA. (A) Mid-IR plasmonic biosensing with graphene-based tunable metasurfaces. (B) Imaging-based molecular barcoding with BIC dielectric metasurfaces. (C) Resolving molecule fingerprint information in dynamic processes with multi-resonant metasurfaces. (D) 3D nanofin plasmonic metasurface with tailored light–matter coupling. (E) Ultrasensitive molecular sensing based on synthesized complex-frequency excitations. (F) Dual-gradient BIC-based metasurfaces with continuous spectral and coupling-strength encoding. (A) Reprinted with permission from [20]. Copyright 2015 American Association for the Advancement of Science. (B) Reprinted with permission from [15]. Copyright 2018 American Association for the Advancement of Science. (C) Reprinted with permission from [194]. Copyright 2018 Springer Nature. (D) Reprinted with permission from [195]. Copyright 2022 American Association for the Advancement of Science. (E) Reprinted with permission from [196]. Copyright 2024 Springer Nature. (F) Reprinted with permission from [197]. Copyright 2024 Springer Nature.

Different from the refractive sensing strategy, molecular fingerprints enhanced and characterized by surface-enhanced infrared absorption (SEIRA) have been attracting more attention [15,16,20,192–198]. For example, Rodrigo et al. [20] demonstrated a high-sensitivity tunable metasurface to chemically specify label-free detection of protein monolayers by exploiting the electro-optical properties of graphene. The resonance frequency of nanostructured graphene is dynamically modulated to cover the wavelength from 1,200 to 2,000 cm^−1^, enabling to discern various protein fingerprints (Fig. 8A). Inspired by BIC physics, Tittl et al. [15] constructed pixelated dielectric metasurfaces with ultrasharp resonances ranging from 1,350 to 1,750 cm^−1^, for enhancing, detecting, and differentiating the absorption fingerprints of various molecules. By comparing the imaging-based barcode differences before and after the coating of bioanalytes, the specific molecule information can be easily read out, as shown in Fig. 8B. Rodrigo et al. [194] leveraged a multi-resonant plasmonic metasurface to enhance vibrational fingerprints of different bioanalytes by providing up to 1,000-fold electric enhancement (Fig. 8C). The interactions of lipid membranes with polypeptides have been successfully monitored in real time. To overcome the low *Q* factors of plasmon resonances, Aigner et al. [195] constructed 3-dimensional (3D) plasmon nanofin metasurfaces using laser nanoprinting technologies. High *Q* factor up to 180 has been achieved under normal incidence. By adjusting the out-of-plane symmetry of the nanofin, the under-coupled, critical, and over-coupled regimes can be flexibly accessed, which is suitable for pixelated molecular sensing (Fig. 8D). Recently, based on synthesized complex-frequency waves, Zeng et al. [196] demonstrated a new strategy to achieve ultrahigh-sensitive molecular sensing, which can amplify the bioanalyte signals by at least an order of magnitude. The enhancement factor could reach 15 when detecting 1.2-nm thickness of monolayer, as shown in Fig. 8E. To further flexibly control light–matter interaction within SEIRA, Aigner et al. [197] introduced a nanophotonic strategy to simultaneously encode the spectral and *Q* factor parameters in 650 × 650 μm^2^ area. Dual-gradient metasurface has been constructed by elaborately designing 27,500 modes to achieve the maximum sensitivity regardless of bioanalyte concentration (Fig. 8F). The comparison of refractive sensing strategy and SEIRA is listed in Table.

**Table.** Comparison of refractive sensing strategy and SEIRA

	Sensitivity	Specificity	Detection target	Working band
Refractive sensing	∆λ/∆n	Extra modification	Bioanalytes with micro and nano sizes	THz, IR, optics
SEIRA	Log(*I*_S_/*I*_0_)	Fingerprint	Macromolecules, organic molecules	THz, mid-IR

### Biofunction techniques

**Fig. 9.**
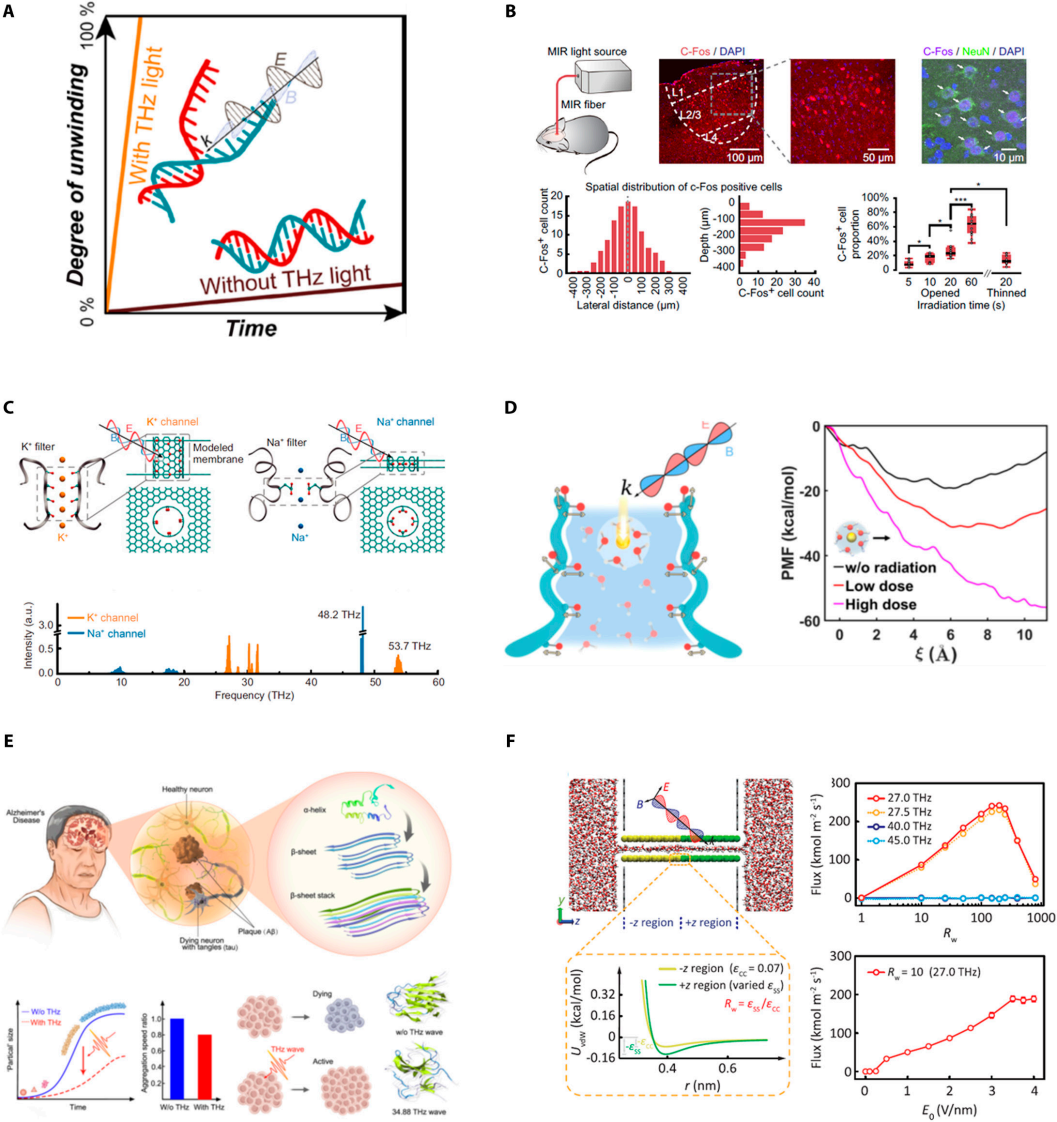
IRT biofunction techniques. (A) IRT wave accelerates DNA unwinding. (B) IRT stimulation activates cortical neurons and accelerates associative learning. (C) IRT stimulation controls neuronal signaling and behavior. (D) IRT wave enhances permeability of the voltage-gated calcium channel. (E) IRT wave disrupts Alzheimer’s β-amyloid fibril formation. (F) Ultrahigh-flux water nanopumps constructed by IRT stimulation. (A) Reprinted with permission from [26]. Copyright 2020 American Chemical Society. (B) Reprinted with permission from [27]. Copyright 2021 Springer Nature. (C) Reprinted with permission from [25]. Copyright 2021 National Academy of Sciences. (D) Reprinted with permission from [23]. Copyright 2021 American Chemical Society. (E) Reprinted with permission from [199]. Copyright 2023 Springer Nature. (F) Reprinted with permission from [28]. Copyright 2024 American Physical Society.

Benefiting from the molecular absorption fingerprint spectrum, recent research has proven that characteristic frequency infrared-terahertz (IRT) waves can nonthermally and reversibly serve as a form of biophysical modulation, such as accelerating DNA regulating, enhancing ion channel permittivity, controlling neuronal signaling, and constructing interface channel [23–28,199,200]. For example, Wu et al. [26] demonstrated that 44-THz IRT stimulus can serve as a long-range method to accelerate DNA duplex unwinding, with a faster speed that increased by 20 times than thermal method, as shown in Fig. 9A. This outstanding research provides a promising avenue to rapidly detect nucleic acids, biomedicine, and therapy. Neuromodulation methods have attracted extensive attention from scientists and clinicians for a long time. IRT stimulation is an emerging technique to modulate neuron regulation and holds promise in clinical applications. Zhang et al. [27] experimentally demonstrated that 53.6-THz IRT stimulus can significantly induce neuron firing activities in the cortical area and accelerate mouse learning speed (50% faster) than control groups (Fig. 9B). Liu et al. [25] showed that 53.6-THz IRT stimulus exerts efficient modulation on neuronal signaling and sensorimotor behaviors (Fig. 9C). Action potential waveform and spiking activity are regulated through nonlinear interactions between IRT and chemical bond vibration at K^+^ channel sieves. Besides, Ca^2+^ ions are closely related with many vital biological processes like cell proliferation and muscle contraction. Li et al. [23] demonstrated that 42.55-THz IRT stimulus can resonate with the stretching mode of either the −COO^−^ or −C=O group (Fig. 9D), which negatively grows nearly 5-fold in the Ca^2+^ free energy. Recently, 34.88-THz waves have been applied to serve as a nonthermal denaturation technique to delay the fibrotic process by 80%, which may provide new method for the remission of related diseases including Alzheimer’s and Parkinson’s disease (Fig. 9E) [199]. Based on molecular dynamic simulation, it is reported that 27-THz IRT stimulus enables unidirectionally transporting massive water through asymmetric wettability membrane channels and construct an ultrahigh-flux nanopump (Fig. 9F), which provides new strategy to design microfluid lab-on-a-chip devices [28].

## Conclusion and Perspectives

### Conclusion

In conclusion, we have highlighted recent advances in nanoengineered THz technologies, focusing on wave generation, modulation, and bio-applications. We have examined state-of-the-art methods for THz generation, spanning the THz region and extending into the IR regime, using heterostructures, metasurfaces, and hybrid systems, highlighting their unique properties and functionalities. We have gone through significant progress in wave modulation techniques, including homogeneous modulation of amplitude, phase, and polarization, as well as individual modulation via spatial light modulators. Additionally, we discussed a series of milestone works in THz biosensing and biophysical modulation, where the THz and IR regimes have shown significant potential in noninvasive imaging, molecular fingerprint characterization, and the regulation of neuronal and biological behaviors. These advances emphasize the versatility of nanoengineered metasurfaces and hybrid structures as foundational tools for enhancing THz technology capabilities.

### Challenges

Despite the progress, several challenges remain. A primary issue is the trade-off between conversion efficiency, bandwidth, and the flexible manipulation of radiation in nanoengineered THz generation techniques. While heterostructure-based STEs demonstrate excellent generation efficiency across nearly the entire THz region and into the IR, their ability to manipulate THz radiation is limited compared to MTEs. MTEs offer significant flexibility in spatiotemporal manipulation and can achieve high-resolution control of THz radiation; however, their conversion efficiency is relatively low, and the bandwidth is often limited, particularly for plasmonic MTEs. Additionally, the intricate and often expensive fabrication processes required for metasurfaces pose challenges to scalability and compatibility with system integration in real-world applications. On the other hand, THz modulation has seen significant improvements, achieving high-speed modulation across a broad bandwidth, while maintaining low loss remains difficult. In biological applications, the main hurdles include how to achieve high-sensitivity biosensing analytes at very low concentrations (often below pg/ml) while ensuring detection specificity and accuracy. Besides, how to further explore the nonthermal biological effects of THz waves and carry out practical applications is still an unresolved topic.

### Perspectives

Looking ahead, several key areas offer exciting potential for further advancements.

For THz generation, while various works have studied the THz emission process from heterostructures and metasurfaces, the role of different physical mechanisms leading to THz emission is not yet fully understood, necessitating further investigation to disentangle these contributions to enhance the THz generation process while maintaining broad bandwidth. One primary opportunity arises in system integration: The exploration of hybrid THz emitters that deeper combine heterostructure and metasurface may promote highly efficient and flexible THz emission and manipulation. Additionally, material innovation presents another avenue for advancement, with the exploration of new materials such as topological insulators [201,202], quantum materials [203], and nanostructures, potentially improving the efficiency and functionality of THz systems. Furthermore, achieving high-resolution, vectorial light field manipulation in both far and near fields could significantly advance THz imaging and spectroscopy. Continued improvements in micro- and nanofabrication techniques, such as nanoscale 3D printing, are also expected to facilitate production scaling while reducing fabrication complexity and costs.

For THz modulation, although metasurfaces have been integrated with various active materials to achieve amplitude modulation of THz waves, there remains a pressing need for advancements that enable the simultaneous attainment of substantial modulation depth, broad modulation bandwidth, and rapid modulation speed to meet the demands of practical applications. Furthermore, previous studies have demonstrated challenges in achieving phase modulation that spans the full 2π range, indicating a necessity for innovative resonant structures to effectively engineer the phase of THz waves. Additionally, in the context of phase and polarization modulation of THz waves, significant fluctuations in amplitude have been observed. Consequently, the development of THz phase and polarization modulators that do not induce amplitude variation is imperative for future research, necessitating novel design strategies for metasurfaces, potentially leveraging deep learning techniques. Moreover, although recent advancements have led to the realization of THz spatial light modulators capable of independently controlling the amplitude and phase of each unit cell, these devices currently operate within a narrow frequency range, which must be expanded in the future. Electrically programmable THz spatial light modulators hold promise for practical applications. However, it is important to note that the THz response of adjacent structural unit is influenced when the amplitude or phase of a single structural unit is manipulated, highlighting the critical need to minimize crosstalk in the future design of THz spatial light modulators.

For THz biosensing, the combination of THz refractive sensing, fingerprint information, and deep learning methods may provide a novel avenue to achieve practical detection in the clinic. Reasonable analysis of large number of biosensing data will provide technical support for ensuring sensitivity, specificity, and reliability. For THz biofunction modulation, it will be necessary to explore new THz sources, detectors, and fiber bundle coupling technologies to make the biophysics mechanism and the medical prospect clearer.

In conclusion, while challenges remain, the extraordinary progress in nanoengineered THz technologies suggests a promising future where advanced THz applications in sensing, imaging, and biomedical fields will be realized on a broad scale. Further interdisciplinary research combining materials science, nanoengineering, and biological sciences will be crucial in unlocking the full potential of these promising technologies.
